# Stenting of Variant Left Carotid Artery Using Brachial Artery Approach in a Patient with Unusual Type of Bovine Aortic Arch

**DOI:** 10.1155/2016/9512318

**Published:** 2016-01-28

**Authors:** Emre Gürel, Zeki Yüksel Günaydın, Ahmet Karagöz, Osman Bektaş, Adil Bayramoğlu, Abdullah Çelik, Aslı Vural

**Affiliations:** ^1^Department of Cardiology, Ordu State Hospital, 52200 Ordu, Turkey; ^2^Department of Cardiology, Ordu University Hospital, 52200 Ordu, Turkey; ^3^Department of Cardiology, Giresun University Hospital, 28200 Giresun, Turkey; ^4^Department of Cardiovascular Surgery, Giresun University Hospital, 28200 Giresun, Turkey

## Abstract

Bovine aortic arch is the most frequently encountered variation in human aortic arch branching. A 63-year-old Asian male presented with symptomatic severe stenosis of left carotid artery originating from the brachiocephalic trunk. Selective engagement to the left carotid artery was unsuccessful using transfemoral approach. We reported on a successful left carotid artery stenting case using right brachial artery approach in a bovine aortic arch. This paper is worthy of reporting in terms of guiding physicians for interventional procedures in these types of challenging cases.

## 1. Introduction

“Bovine aortic arch” refers to the most common variant of aortic arch branching in humans. This configuration can be found in approximately 27% of the population and can be further divided into 2 subtypes. In the less usual subtype (7%), the left common carotid artery (CCA) originates directly from the innominate artery, while the more usual subtype can be described as the common origin of the innominate artery and the left CCA and can be found in approximately 20% of the population [1]. However, the actual bovine arch pattern, found in cattle, is different and has no resemblance to any of the above described human aortic arch patterns. A single great vessel originates from the aortic arch which gives two subclavians for either side and a bicarotid trunk. The bicarotid trunk then divides into the left and right CCA [2].

In these anatomic variants, selective cannulation of the left CCA is often technically difficult from the transfemoral approach. We report on a successful left carotid stenting case using right brachial artery approach in a patient with “bovine aortic arch.”

## 2. Case Presentation

**Figure 1:**
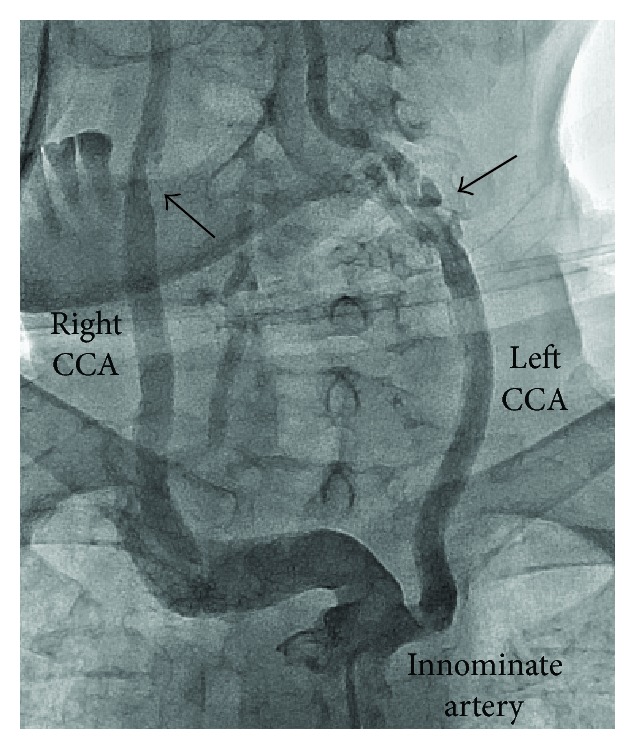
Aortic arch angiogram revealed critical stenosis in the proximal right internal carotid artery (black arrow), variant origin of the left common carotid artery from the innominate artery, and also critical stenosis in the proximal left internal carotid artery (black arrow) (CCA: common carotid artery).

**Figure 2:**
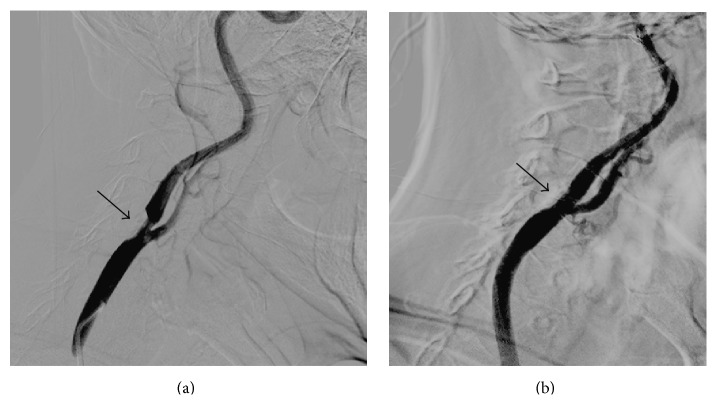
Selective right carotid angiography confirmed critical stenosis (black arrows) in the right internal carotid artery at the bifurcation level (a). Successful carotid stent placement was performed without residual stenosis (b).

A 63-year-old Asian male with hypertension was admitted due to recent attacks of transient right upper extremity weakness and numbness, lasting 3 to 5 min. On admission, his blood pressure was high at 170/90 mmHg and heart rate was 80 beats/min. Neurologic examination was normal. Doppler ultrasound examination showed normal right internal carotid artery velocities (0.92 m/s of peak systolic velocity) and elevated velocities in the left internal carotid artery (2.79 m/s of peak systolic velocity) suggesting critical stenosis. Six months before this admission, he had undergone arch aortography at an outside institution using a femoral artery approach, which confirmed critical stenosis in the proximal right internal carotid artery (ICA), variant origin of the left CCA from the proximal brachiocephalic trunk, and also critical stenosis in the proximal left ICA (Figure 1). After selective cannulation of right CCA, successful carotid stent placement had been performed with distal protection at the bifurcation level (Figure 2). Left carotid stenosis had not been stented at that time. Because of the right sided transient ischemic attack episodes on admission to our institution, we decided to perform stenting to the stenosis in left ICA. Firstly, femoral approach was tried. However, selective engagement to the left CCA could not be possible in this way using standard catheters and Simmons catheter; therefore, right brachial approach was considered.

**Figure 3:**
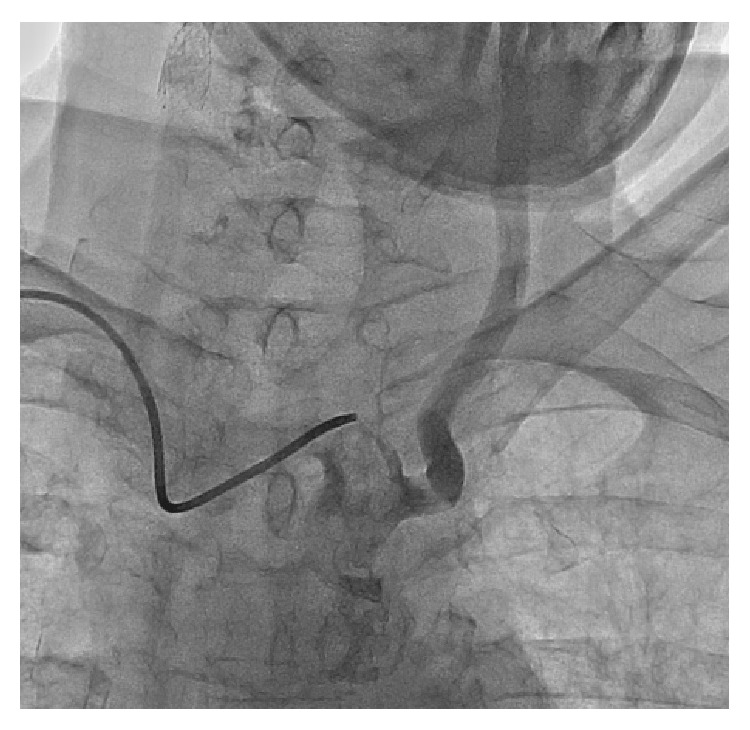
A 5 Fr hydrophilic coated vertebral catheter was advanced into the brachiocephalic trunk to engage selectively the left common carotid artery.

**Figure 4:**
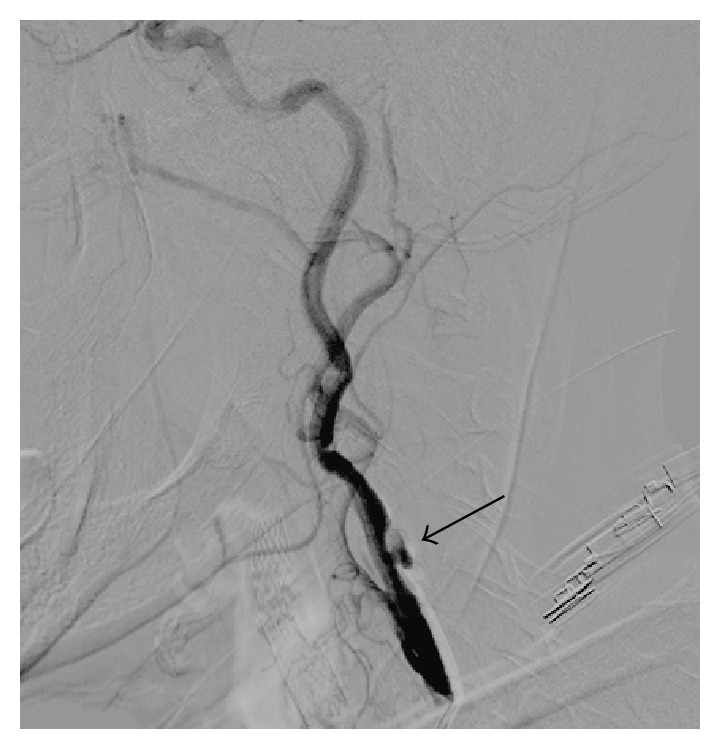
Selective left carotid angiography confirmed critical stenosis in the left internal carotid artery at the bifurcation level (black arrow).

**Figure 5:**
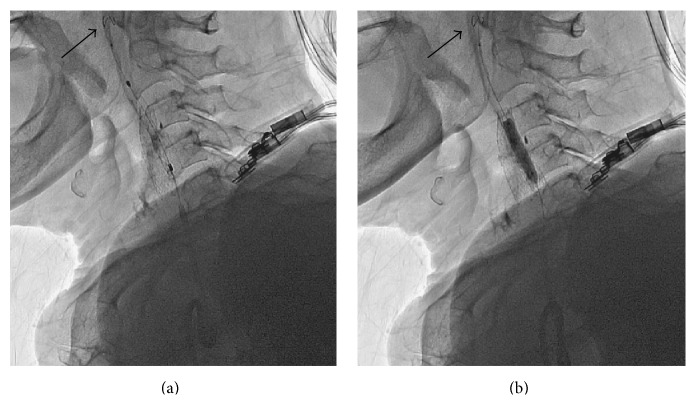
Self-expandable stent deployment (a) and postdilation (b) was performed after placement of the distal embolic protection system (black arrows) in the left internal carotid artery.

**Figure 6:**
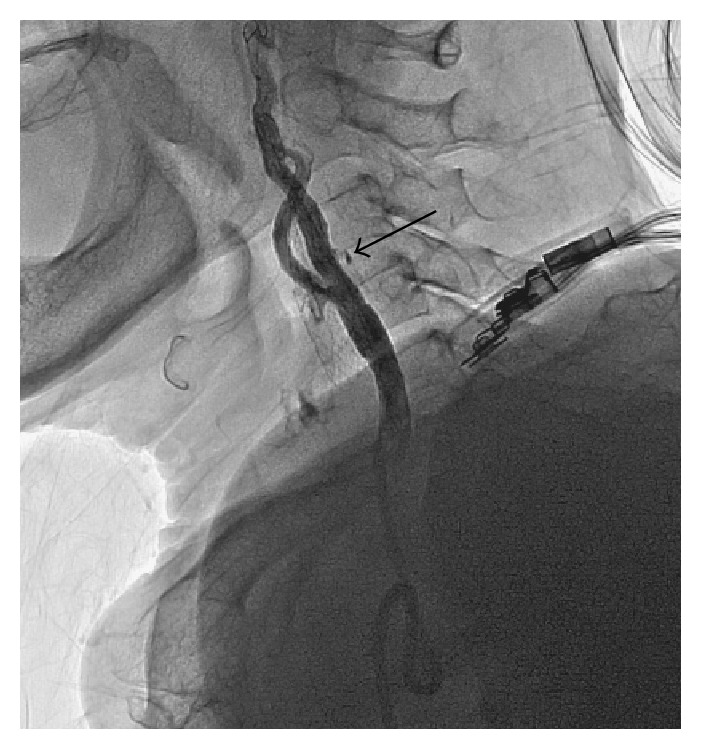
Left carotid angiography after stent deployment shows no residual stenosis in the internal carotid artery (black arrow).

After pretreatment with aspirin (325 mg daily) and clopidogrel (75 mg daily) for five days, a 7 Fr sheath was inserted in the right brachial artery. A 5 Fr hydrophilic coated vertebral catheter (Merit Medical Systems, Inc., Utah, USA) was then advanced into the brachiocephalic trunk to engage selectively the left CCA (Figure 3). 0.035′′ flexible guidewire (Merit Medical Systems, USA) was positioned at the left external carotid artery using this vertebral catheter and exchanged with a 0.035′′ Amplatz extra-support guidewire. After that, a 7 Fr Asahi Zenyteex guiding catheter (Asahi-Intecc, USA) was positioned at the left common carotid artery and extra-support guidewire was removed. Selective left carotid angiography confirmed critical stenosis at the bifurcation level of ICA (Figure 4). Distal embolic protection system (Emboshield, Abbot, IL, USA) was deployed in the distal ICA under fluoroscopic guidance. A 6 × 9 × 40 mm Sinus Carotid (Optimed, Ettlingen, Germany) self-expandable stent was successfully deployed in the left common and internal carotid artery, and postdilation was performed using a 5 × 20 mm Pyxis-c balloon (Stron Medical, Winsen, Germany) with the inflation at 10 atm for 10 sec (Figure 5). After postdilation, the follow-up angiogram showed complete coverage of the lesion without residual stenosis or dissection (Figure 6). The embolic protection system was removed and moderate debris was identified. The brachial artery sheath was removed and excellent hemostasis was achieved. The patient tolerated the procedure well and did not suffer any complications. He was discharged within 24 hours without any neurologic deficit and is now followed up at the outpatient clinic in a stable condition.

## 3. Discussion

Percutaneous carotid artery stenting represents a minimally invasive and less traumatic alternative to conventional surgical repair for managing carotid artery stenosis in selected patients, particularly those with younger (less than 70 years) and/or higher surgical risk [3, 4]. However, it is reported that transfemoral intervention is difficult in 1-2% of cases due to occlusive disease of the aorta and the iliofemoral arteries [5]. Alternative vascular access routes, such as the brachial artery, radial artery, or the direct route using the carotid artery, have been used in these cases [6–8]. Recent experiences with transbrachial carotid procedures have demonstrated that the brachial artery is a safe, feasible, and useful access route when the transfemoral procedure is difficult [9]. In our patient, the origin of the left CCA from the brachiocephalic trunk made the femoral approach less desirable. This bovine variant can be seen in 7% of the general population and is distinct from the more common bovine arch configuration that occurs in 27% involving a common origin of the brachiocephalic trunk and the left common carotid artery from the aortic arch. Although we used Simmons catheter containing complex curves in addition to standard catheters, selective cannulation of the left CCA could not be possible. For this reason, brachial access was considered. While performing the brachial approach, the guiding catheter position was stable and allowed the successful introduction of the balloon catheter and stent. This case showed that the right brachial access might be useful alternative for stenting of variant left carotid artery in patients with unusual type of bovine aortic arch. Carotid artery angioplasty and stenting procedures are technically difficult from the transfemoral approach in variant aortic arch branching such as “bovine aortic arch.” We performed a right brachial artery approach for selective cannulation of a variant left CCA originating from the brachiocephalic trunk and performed successful carotid stenting with distal protection.
